# Rapid, efficient, and simple motor neuron differentiation from human pluripotent stem cells

**DOI:** 10.1186/s13041-015-0172-4

**Published:** 2015-12-01

**Authors:** Daisuke Shimojo, Kazunari Onodera, Yukiko Doi-Torii, Yasuharu Ishihara, Chinatsu Hattori, Yukino Miwa, Satoshi Tanaka, Rina Okada, Manabu Ohyama, Masanobu Shoji, Atsushi Nakanishi, Manabu Doyu, Hideyuki Okano, Yohei Okada

**Affiliations:** Department of Neurology, Aichi Medical University School of Medicine, Aichi, 480-1195 Japan; Department of Physiology, Keio University School of Medicine, Tokyo, 160-8582 Japan; Department of Neurology, Nagoya University Graduate School of Medicine, Nagoya, 466-8550 Japan; Department of Orthopedic Surgery, Nagoya University Graduate School of Medicine, Nagoya, 466-8550 Japan; Division of Regenerative Medicine, Jikei University School of Medicine, Tokyo, 105-8461 Japan; Department of Dermatology, Keio University School of Medicine, Tokyo, 160-8582 Japan; Integrated Technology Research Laboratories, Pharmaceutical Research Division, Takeda Pharmaceutical Company Limited, Kanagawa, 251-8555 Japan

**Keywords:** Human embryonic stem cells, Human induced pluripotent stem cells, Motor neurons, Long-term culture of motor neurons, Lentiviral reporter

## Abstract

**Background:**

Human pluripotent stem cells (hPSCs) are being applied in regenerative medicine and for the *in vitro* modeling of human intractable disorders. In particular, neural cells derived from disease-specific human induced pluripotent stem cells (hiPSCs) established from patients with neurological disorders have been used as *in vitro* disease models to recapitulate *in vivo* pathogenesis because neural cells cannot be usually obtained from patients themselves.

**Results:**

In this study, we established a rapid, efficient, and simple method for efficiently deriving motor neurons from hPSCs that is useful for pathophysiological analysis and the development of drugs to treat motor neuron diseases. Treatment with GSK3β inhibitors during the initial phase of differentiation in combination with dual SMAD inhibition was sufficient to induce *PAX6*^+^ and *SOX1*^+^ neural progenitors within 1 week, and subsequent treatment with retinoic acid (RA) and purmorphamine, which activates sonic hedgehog (SHH) signaling, resulted in the highly efficient induction of HB9^+^ and ISL-1^+^ motor neurons within 2 weeks. After 4 weeks of monolayer differentiation in motor neuron maturation medium, hPSC-derived motor neurons were shown to mature, displaying larger somas and clearer staining for the mature motor neuron marker choline acetyltransferase (ChAT). Moreover, hPSC-derived motor neurons were able to form neuromuscular junctions with human myotubes *in vitro* and induced acetylcholine receptor (AChR) clustering, as detected by Alexa 555-conjugated α-Bungarotoxin (α-BTX), suggesting that these hPSC-derived motor neurons formed functional contacts with skeletal muscles. This differentiation system is simple and is reproducible in several hiPSC clones, thereby minimizing clonal variation among hPSC clones. We also established a system for visualizing motor neurons with a lentiviral reporter for HB9 (*HB9*^*e438*^::*Venus*). The specificity of this reporter was confirmed through immunocytochemistry and quantitative RT-PCR analysis of high-positive fractions obtained via fluorescence-activated cell sorting (FACS), suggesting its applicability for motor neuron-specific analysis.

**Conclusions:**

Our motor neuron differentiation system and lentivirus-based reporter system for motor neurons facilitate the analysis of disease-specific hiPSCs for motor neuron diseases.

**Electronic supplementary material:**

The online version of this article (doi:10.1186/s13041-015-0172-4) contains supplementary material, which is available to authorized users.

## Background

Human pluripotent stem cells (hPSCs), including human embryonic stem cells (hESCs) and human induced pluripotent stem cells (hiPSCs), can be differentiated into all cell types of the human body and have been applied in regenerative medicine and the pathophysiological analysis of intractable disorders [1]. In particular, neural cells derived from disease-specific hiPSCs from patients with neurological disorders have been especially useful as *in vitro* disease models recapitulating *in vivo* pathogenesis, as cells in the nervous system cannot be usually obtained from patients themselves.

Amyotrophic lateral sclerosis (ALS), spinal muscular atrophy (SMA), and spinal and bulbar muscular atrophy (SBMA) are motor neuron diseases. Although these motor neuron diseases exhibit different molecular pathologies, they share a common phenotype: motor neuron degeneration. To reveal the mechanisms underlying motor neuron degeneration and to develop novel drugs, researchers have taken advantage of motor neurons derived from disease-specific hiPSCs for pathological analysis [2–4]. However, the methods reported for motor neuron derivation from hPSCs in previous studies are time-consuming and require complicated manipulations. Moreover, the efficiency of these methods tends to be low, and show variability depending on hPSC clones referred to as clonal variations [5].

In this study, we established a simple, rapid, and reproducible method for efficiently deriving motor neurons from hPSCs without the transduction of any exogenous genes. This method facilitates simple and accurate pathophysiological analysis of motor neuron diseases using disease-specific hiPSCs.

## Results

### Rapid and efficient motor neuron differentiation from human pluripotent stem cells

**Fig. 1:**
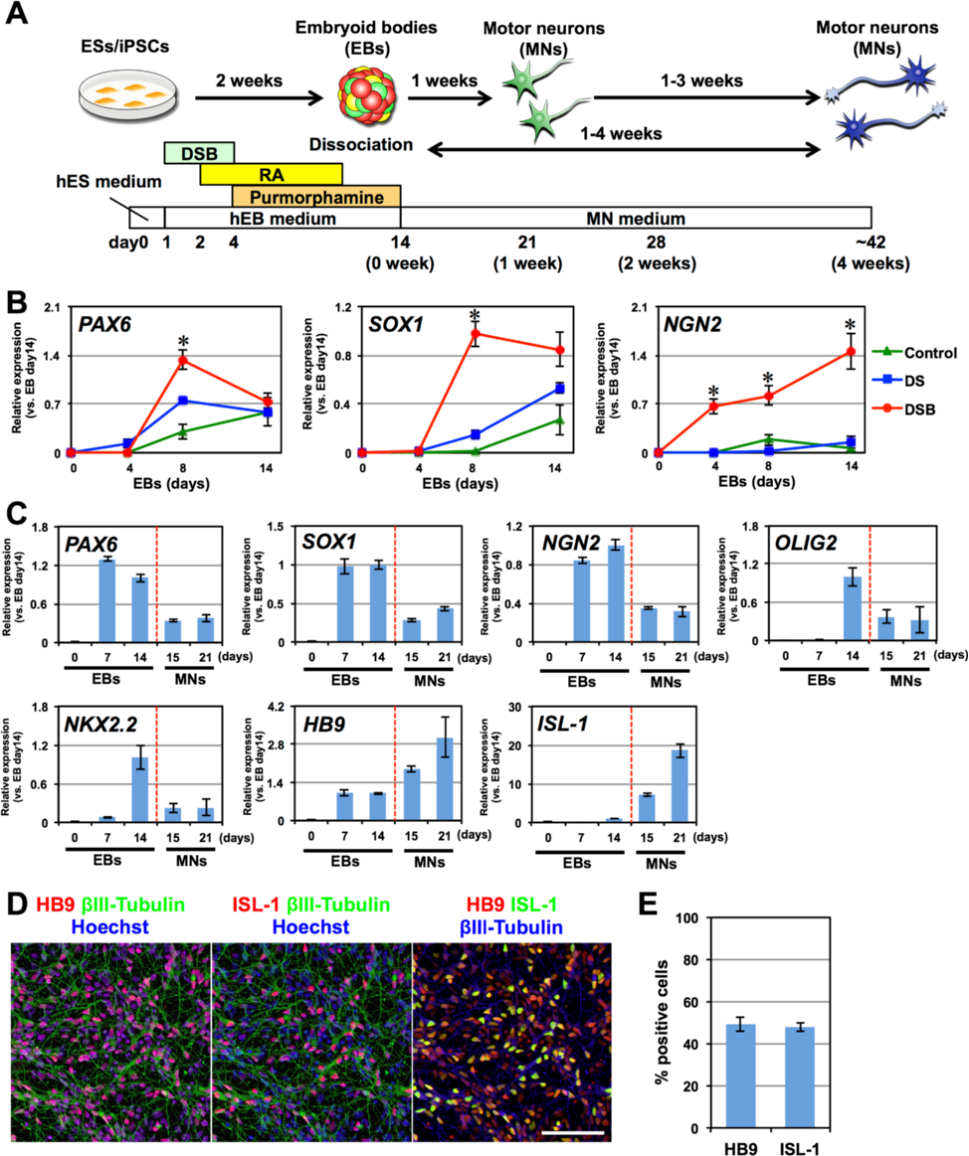
Rapid, efficient and simple motor neuron differentiation from human pluripotent stem cells. **a** Schematic presentation of motor neuron differentiation from hPSCs. **b** Time-course analysis of the expression of *PAX6*, *SOX1*, and *NGN2* in EBs via quantitative RT-PCR analysis. Control, 0.09 % DMSO. DS, dorsomorphin and SB431542. DSB, dorsomorphin, SB431542 and BIO. *n* = 4, mean ± SEM. *, *p* < *0.05* (Student’s t test). **c** Time-course analysis of the expression of *PAX6*, *SOX1*, *NGN2*, *OLIG2*, *NKX2.2*, *HB9*, and *ISL*-*1* mRNA in EBs and during monolayer motor neuron (MN) differentiation. *n* = 4, mean ± SEM. **d** Immunocytochemical analysis of HB9, ISL-1, and βIII-Tubulin 1 week after monolayer differentiation. Scale bar, 100 μm. **e** Quantitative analysis of the number of the HB9^+^ and ISL-1^+^ cells. *n* = 3, mean ± SEM

By modifying our previously established method for deriving neural stem/progenitor cells (NS/PCs) as neurospheres from hPSCs through embryoid body (EB) formation [6, 7], we established a rapid neural differentiation protocol from hPSCs (Fig. 1a). Because the derivation of NS/PCs from EBs using the previously established method takes 1 month, we first utilized dual SMAD inhibition to facilitate the neural differentiation of EBs [8]. KhES1 human embryonic stem cells [9] were detached from the feeder layer *en bloc* and cultured in suspension to form EBs using a BMP inhibitor (3 μM dorsomorphin) and a TGFβ inhibitor (3 μM SB4315342) during the first three days of differentiation (DS) (from day 1 to day 4). Although this dual SMAD inhibition slightly increased the expression of neural markers (*PAX6* and *SOX1*) compared with untreated control cells, it was not sufficient to derive neural progenitors from EBs within two weeks of differentiation (Fig. 1b). Thus, to further facilitate neural differentiation, we added the GSK3β inhibitor (3 μM (2′Z, 3′E)-6-bromoindirubin-3′-oxime (BIO)) during the same period, in addition to dual SMAD inhibition (DSB). As a result, on day 8, the expression of *PAX6* and *SOX1* was significantly increased compared with control cells, by 3.7 ± 0.4-fold and 138 ± 34-fold, respectively (Fig. 1b). Moreover, the expression of the proneural gene *NGN2* was increased by 68 ± 16-fold after 14 days of differentiation compared with untreated control cells. These results suggest that GSK3β inhibition has a positive effect on the neural differentiation of hESCs in our differentiation system, possibly by activating the canonical Wnt pathway through β-catenin. To drive differentiation into motor neurons, we added retinoic acid (RA) (from day 2 of EB formation) and purmorphamine, which activates the sonic hedgehog (SHH) signaling pathway (from day 4 of EB formation), to confer caudal and ventral regional identities, respectively, upon hESC-derived NS/PCs. On day 14 of differentiation, the EBs expressed not only markers for the neural progenitors *PAX6* and *SOX1* but also additional transcription factors expressed in motor neuron progenitors, including *OLIG2* and *NGN2* (Fig. 1c). Subsequently, the EBs were dissociated into single cells and were adherently differentiated into neurons via monolayer culture in motor neuron medium (MNM). Within 1 week of adherent differentiation, *HB9* and *ISL*-*1*, markers for somatic motor neurons, were significantly upregulated (Fig. 1c). Consistent with these results, immunocytochemical analysis revealed the efficient induction of HB9^+^/ISL-1^+^/βIII-Tubulin^+^ motor neurons on day 7 of monolayer differentiation (HB9, 49.1 ± 3.3 %, ISL-1, 47.9 ± 2.0 %, *n* = 3, Fig. 1d, e). Thus, we have established a rapid, efficient, and simple culture protocol that achieves motor neuron differentiation in only 2 weeks.

### Effects of long-term hESC-derived motor neuron culture *in vitro*

**Fig. 2:**
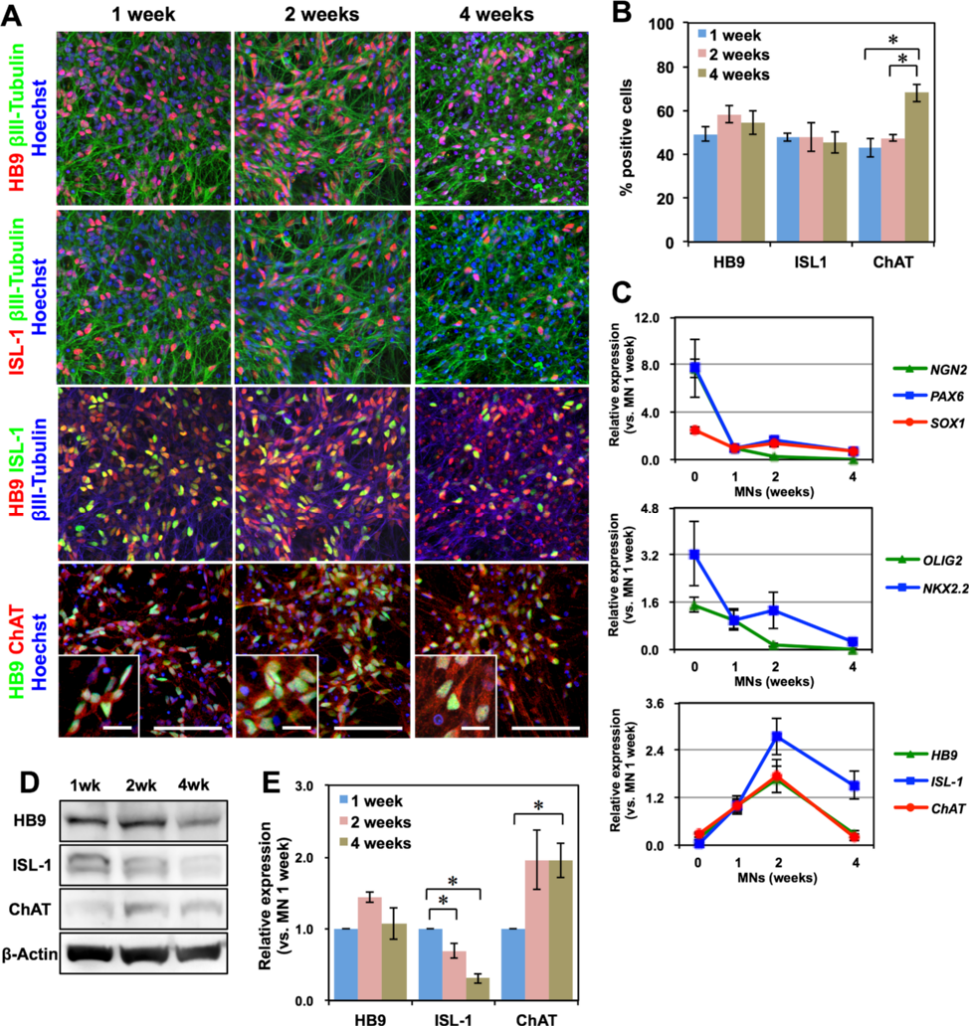
Long-term culture of hESC-derived motor neurons. **a** Immunocytochemical analysis of HB9, ISL-1, βIII-Tubulin, and ChAT after 1, 2, or 4 weeks of monolayer differentiation. Scale bar, 100 μm, and 20 μm (inset). **b** Quantification of the number of the cells positive for each marker. *n* = 4, mean ± SEM. *, p < *0.05* (Student’s t test). **c** Time-course analysis of the expression of *PAX6*, *SOX1*, *NGN2*, *OLIG2*, *NKX2.2*, *HB9*, *ISL*-*1*, and *ChAT* during monolayer differentiation via quantitative RT-PCR. *n* = 4, mean ± SEM. **d** Western blot analysis of the expression of the HB9, ISL-1, and ChAT proteins during monolayer differentiation. **e** Quantitative analysis of the expression of the HB9, ISL-1, and ChAT proteins through densitometry using ImageJ. The protein expression levels are normalized to β-Actin. *n* = 4, mean ± SEM. *, *p* < *0.05*, (Student’s t test)

To investigate the effects of the long-term culture of hESC-derived motor neurons, we examined temporal alterations of the expression of several motor neuron markers during 4 weeks of monolayer differentiation in MNM. Based on immunocytochemical analysis, the proportion of HB9^+^ and ISL-1^+^ cells did not change greatly and consistently comprised approximately 50–60 % of the total cells (Fig. 2a, b). By contrast, the proportion of cells positive for ChAT, a marker of mature motor neurons, was 54.6 ± 4.5 % at the end of one week and increased to 68.1 ± 4.1 % by 4 weeks of monolayer differentiation. Moreover, after 4 weeks of monolayer differentiation, the somas of the motor neurons had grown larger and were stained more clearly for ChAT compared with those observed after 1 week of monolayer differentiation (Fig. 2a, insets). Quantitative RT-PCR analysis revealed that the expression of genes known to be expressed in neural progenitor cells (*PAX6*, *SOX1*, *NGN2*, *OLIG2*, and *NKX2.2*) decreased during 4 weeks of monolayer differentiation, whereas markers of motor neurons (*HB9*, *ISL*-*1*, and *ChAT*) were increased during the first 2 weeks of differentiation, followed by a gradual decrease during the subsequent 2 weeks (Fig. 2c). To address the discrepancy in the time course of motor neuron marker expression between the immunocytochemistry and quantitative RT-PCR analyses, we also performed a western blot analysis (Fig. 2d, e). We found that the expression of the ChAT protein significantly increased during 4 weeks of differentiation, whereas the HB9 protein level was not significantly altered during this period (Fig. 2e). The ISL-1 protein was significantly decreased during monolayer differentiation as observed in the quantitative RT-PCR analysis (Fig. 2c). These results suggest that the expression of HB9 and ChAT might be regulated post-transcriptionally during the culture of hESC-derived motor neurons and that 4 weeks of monolayer differentiation facilitates the maturation of hESC-derived motor neurons.

### Co-culture with myotubes and the formation of neuromuscular junctions

**Fig. 3:**
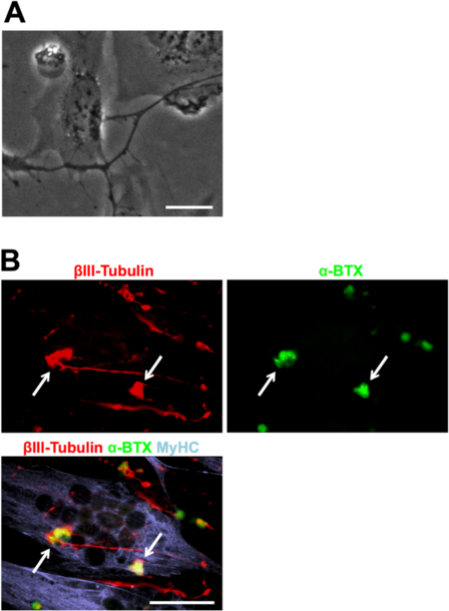
Co-culture of hESC-derived motor neurons with myotubes. **a** Motor neurons derived from KhES1 cells were plated on myotubes derived from the human myoblast cell line Hu5/E18 to form end plate-like structures. Scale bar, 20 μm. **b** Immunocytochemical analysis of βIII-Tubulin and MyHC after 3 days of co-culture with motor neurons and myotubes. AChR clusters were visualized with Alexa 555-conjugated α-Bungarotoxin (α-BTX, white allows). Scale bar, 50 μm

To examine the functional properties of hESC-derived motor neurons, we co-cultured hESC-derived motor neurons with skeletal muscle myotubes derived from the human myoblast cell line Hu5/E18 [10, 11]. KhES1-derived EBs containing motor neuron progenitors were partially dissociated and plated onto human myotubes differentiated from Hu5/E18 in MNM for 3–4 days. hESC-derived motor neurons labeled with βIII-Tubulin contacted myotubes, formed end plate-like structures, and induced clustering of acetylcholine receptors, which was visualized using Alexa 555-conjugated α-Bungarotoxin (Alexa 555-α-BTX) (Fig. 3a, b). These results suggest that motor neurons derived in our system are competent to form functional neuromuscular junctions (NMJs) *in vitro*.

### Differentiation of hiPSCs into motor neurons

**Fig. 4:**
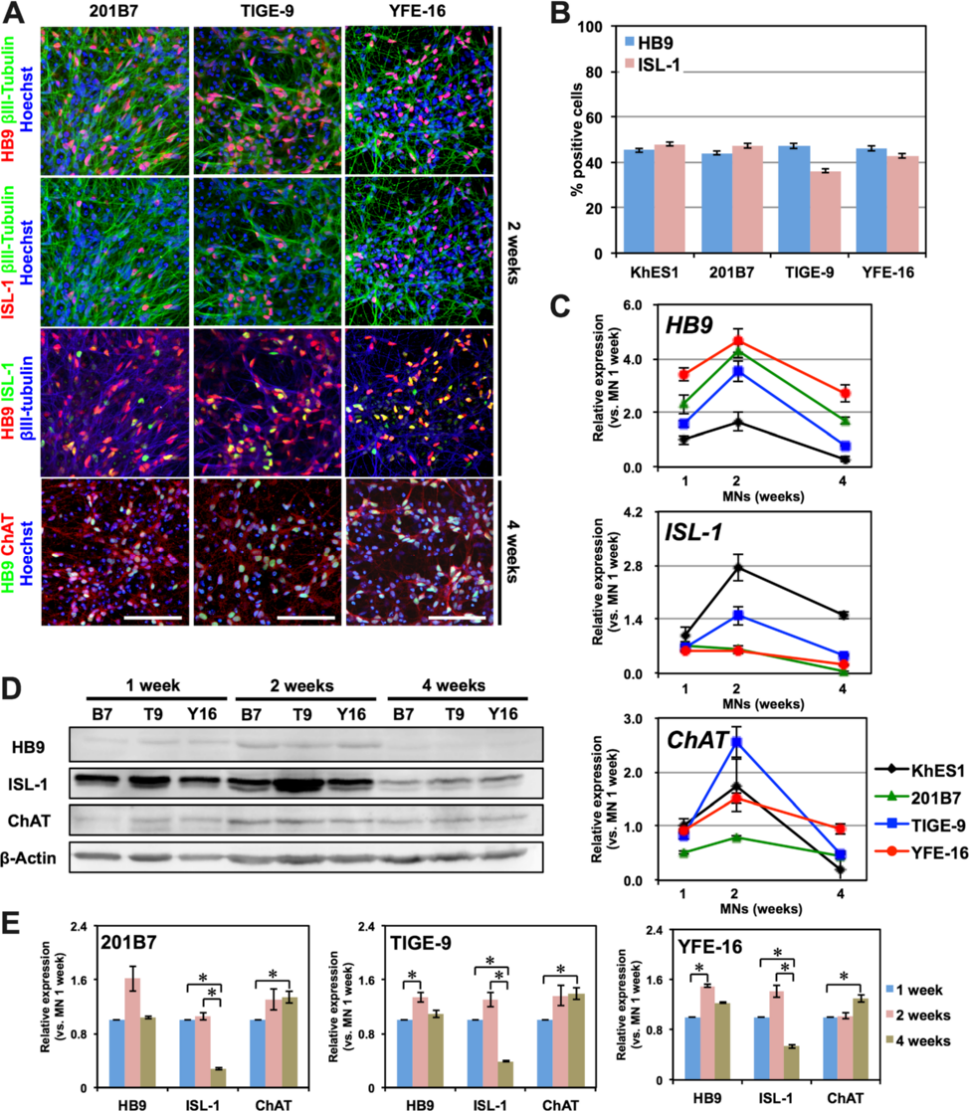
Derivation of motor neurons from hiPSCs. **a** Immunocytochemical analysis of motor neurons derived from 201B7, TIGE-9 and YFE-16 for HB9, ISL-1, and βIII-Tubulin after 2 weeks of monolayer differentiation and ChAT after 4 weeks of monolayer differentiation. Scale bar, 100 μm. **b** Quantitative analysis of the number of the cells positive for motor neuron markers in cultures derived from each hiPSC clone. *n* = 3, mean ± SEM. **c** Time-course analysis of the expression of *HB9*, *ISL*-*1*, and *ChAT* during monolayer differentiation of hiPSC-derived motor neurons via quantitative RT-PCR. *n* = 3, mean ± SEM. **d** Western blot analysis of the expression of the HB9, ISL-1, and ChAT proteins during monolayer differentiation of hiPSC-derived motor neurons. B7, 201B7. T9, TIGE-9. Y16, YFE-16. **e** Quantitative analysis of the expression of the HB9, ISL-1, and ChAT proteins through densitometry using ImageJ. Protein expression levels are normalized to β-Actin. *n* = 3, mean ± SEM. *, *p* < *0.05*, (Student’s t test)

We further examined whether this motor neuron differentiation system is applicable to hiPSCs. Three hiPSC clones (201B7, TIGE-9, and YFE-16) were differentiated into motor neurons following the same protocol (Fig. 4). 201B7 was kindly provided by Dr. Yamanaka [1], and TIGE-9 and YFE-16 were established in our laboratory from fibroblasts from adult males (36 and 24 years of age, respectively) by introducing *OCT4*, *SOX2*, *KLF4*, *L*-*MYC*, *LIN28*, and *shTP53* via episomal vectors; the last two clones were subjected to analysis of pluripotent marker expression via immunocytochemistry and quantitative RT-PCR, silencing of episomal transgenes, karyotype analysis, and teratoma formation capacity testing (Additional file 1: Figure S1). All of these clones efficiently differentiated into HB9^+^ and ISL-1^+^ motor neurons that expressed the mature motor neuron marker ChAT by 4 weeks of monolayer differentiation (Fig. 4a). The proportions of HB9^+^ and ISL-1^+^ cells 1 week after monolayer differentiation of dissociated EBs derived from the three hiPSC clones were similar to those obtained from KhES1 hESCs, at approximately 40-50 % (Fig. 4b). We also examined the time course of motor neuron marker expression via quantitative RT-PCR and western blotting and confirmed similar expression profiles in all of the hiPSC clones to those observed in KhES1 cells (Fig. 4c-e). These results suggest that our differentiation protocol is applicable to hiPSCs, thereby minimizing clonal variation among the hPSC clones.

### Replacement of small-molecule compounds by more specific and less toxic inhibitors

**Fig. 5:**
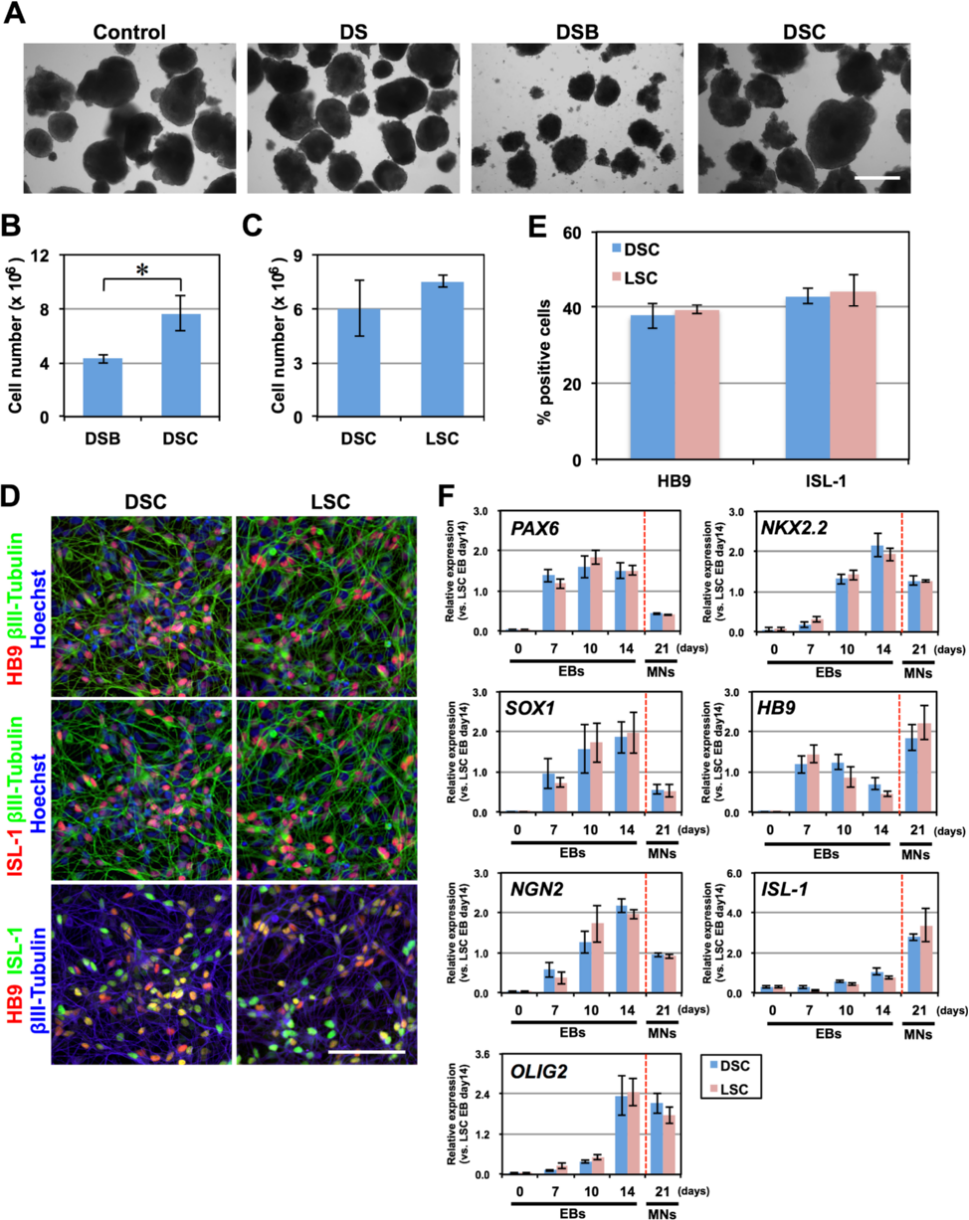
Replacement of small molecule compounds with more specific and less toxic inhibitors. **a** Morphology of EBs, which were derived from 201B7, treated with vehicle (DMSO), DS, DSB, or DSC on day 4. Massive cell death was observed after the treatment of DSB. Scale bar = 200 μm. Control, 0.09 % DMSO. DS, dorsomorphin and SB431542. DSB, dorsomorphin, SB431542, and BIO. DSC, dorsomorphin, SB431542, and CHIR99021. **b** The number of cells harvested from DSB- and DSC-treated EBs on day 14 per 100-mm culture dish of starting hiPSCs. *n* = 4, mean ± SEM. *, *p* < *0.05* (Student’s t test). **c** The number of cells harvested from DSC- and LSC-treated EBs on day 14 per 100-mm culture dish of starting hiPSCs. *n* = 4, mean ± SEM. LSC, LDN193189, SB431542, and CHIR99021. **d** Immunocytochemical analysis of motor neurons derived from DSC- or LSC-treated EBs for HB9, ISL-1 and βIII-Tubulin on day 7 of monolayer differentiation. Scale bar, 100 μm. **d** Quantitative analysis of the number of the cells positive for HB9 and ISL-1. *n* = 4, mean ± SEM. **e** Time-course analysis of the expression of *PAX6*, *SOX1*, *NGN2*, *OLIG2*, *NKX2.2*, *HB9*, and *ISL*-*1* in EBs and during monolayer differentiation of motor neurons (MN) via quantitative RT-PCR. *n* = 4, mean ± SEM

Although our differentiation protocol achieved rapid and efficient differentiation of motor neurons from hPSCs, we observed significant cell death during EB formation after the addition of 3 compounds (DSB), which may have been caused by the cytotoxicity of the small-molecule compounds (Fig. 5a). To avoid this massive cell death and more efficiently obtain motor neuron progenitors for further analysis, we examined the effects of other compounds that inhibit BMP and GSK3β on the differentiation of 201B7 iPSCs. We first replaced BIO with CHIR99021, a more specific and less toxic inhibitor of GSK3β [12], and observed a significant reduction in cell death during EB formation (Fig. 5a). Treatment of EBs with CHIR99021 in combination with DS (DSC) resulted in a 1.79 ± 0.50-fold increase in the cell number obtained from EBs after 14 days of differentiation compared with those treated with DSB (Fig. 5b). We also examined the effects of LDN193189, a more specific and less toxic inhibitor of BMP signals [13]. When EBs were treated with LDN193189, SB4315342, and CHIR99021 (LSC), we observed a 1.54 ± 0.43-fold increase in the number of cells obtained from EBs after 14 days of differentiation compared with those treated with DSC, but the difference was not statistically significant (Fig. 5c). No significant differences in the differentiation of motor neurons were observed among the populations subjected to the DSB, DSC, and LSC treatments. The proportions of HB9^+^ motor neurons under DSC and LSC conditions were 37.9 ± 3.4 and 39.4 ± 1.1 %, respectively (Fig. 5d, e), and the results of time-course analysis of the expression of motor neuron markers did not differ significantly among the DSB, DSC, and LSC treatments (Fig. 4a, b and 5d, e). Together, these results suggest that both the DSC and LSC protocols are useful in practice for obtaining a greater number of motor neuron progenitors and that the inhibition of BMP and TGFβ signals combined with inhibition of GSK3β, which activates canonical WNT signaling, have positive effects on the induction of neural progenitors, excluding the off-target effects of these compounds.

### Visualization of motor neurons using an *HB9*^*e438*^::*Venus* reporter lentivirus

**Fig. 6:**
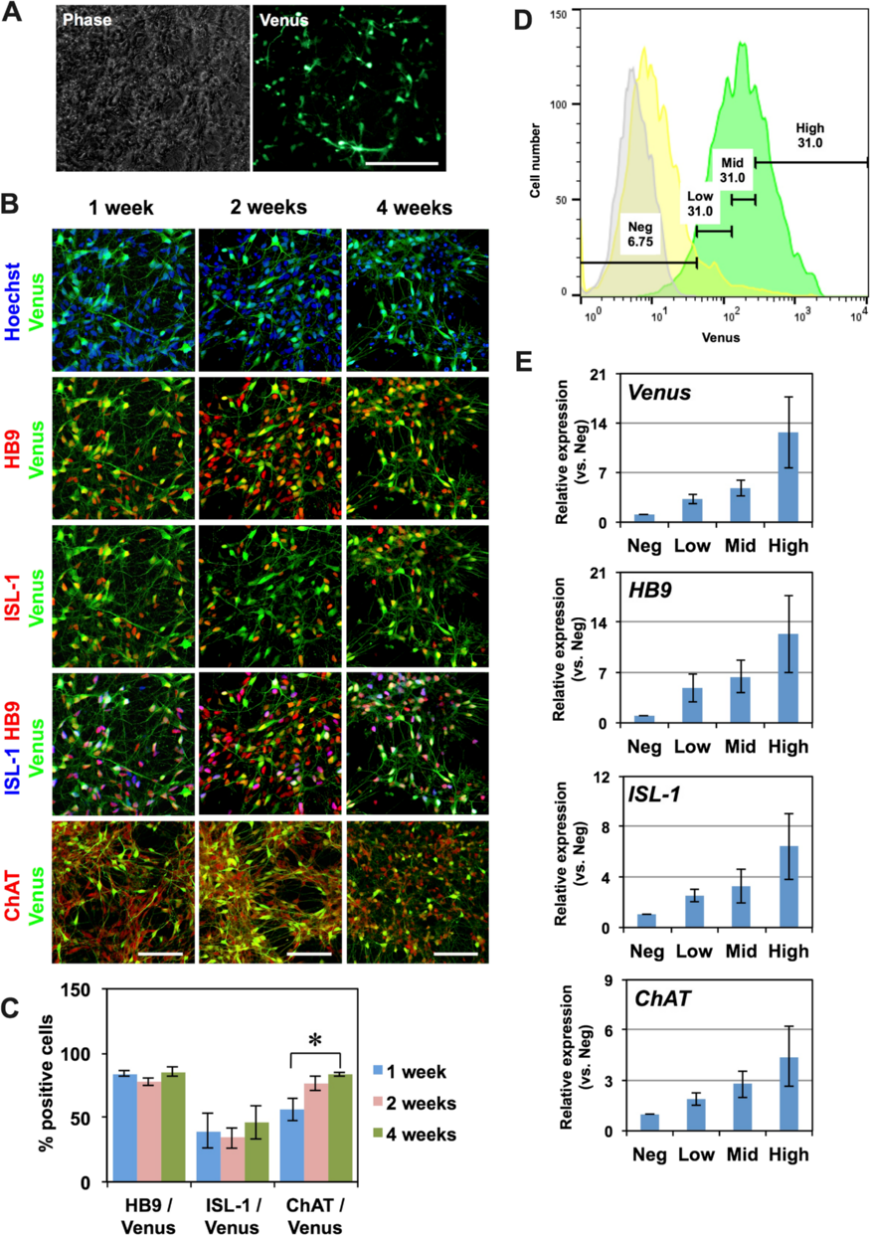
Visualization of motor neurons using the *HB9* ^*e438*^::*Venus* reporter lentivirus. **a** Live-cell imaging of *HB9* ^e438^::Venus^+^ motor neurons 3 days after lentivirus infection. Scale bar, 100 µm. **b** Immunocytochemical analysis of motor neurons for HB9, ISL-1, ChAT, and Venus (GFP antibody) at 1, 2, and 4 weeks after *HB9* ^*e438*^::*Venus* lentivirus infection. Scale bar, 100 μm. **c** Quantitative analysis of the number of cells positive for HB9, ISL-1, and ChAT among Venus^+^ cells. *n* = 4, mean ± SEM.*, *p* < *0.05* (Student’s t test) **d** Histograms of *HB9* ^*483*^::*Venus* lentivirus-infected cells (Green), background lentivirus (*β*-*glo*::*Venus*)-infected cells (Yellow), and uninfected control cells (Gray) via flow cytometry. The *HB9* ^*e438*^::Venus^+^ cells were divided into 4 fractions: a negative fraction (Neg), in which the fluorescence intensities were equivalent to an uninfected negative control, and a low-positive fraction (Low), a middle-positive fraction (Middle), and a high-positive fraction (High), in which the fluorescence intensities were equivalent to the lowest 1/3, the next lowest 1/3, and the highest 1/3 of fluorescence intensities within the positive fraction, respectively. **e** The expression of *Venus*, *ISL*-*1*, *HB9*, and *ChAT* in each fraction determined via quantitative RT-PCR. *n* = 3, mean ± SEM. A significant increase in the expression of *Venus*, *HB9*, and *ISL*-*1* in the high-positive fraction was observed (*p* < *0.05*, Kruskal-Wallis test)

Although our method achieved efficient motor neuron derivation, the proportion of HB9^+^ motor neurons was approximately 40–50 %, and other cell types persisted in culture, presenting an obstacle for the analysis of motor neuron-specific biological events. To address these issues, we constructed an *HB9*^*e438*^::*Venus* reporter lentivirus, in which the Venus fluorescent protein (an altered yellow fluorescent protein; [14]) is expressed under the control of the 438-base pair *HB9* enhancer element (*HB9*^*e438*^, [15]) and the human β-globin minimal promoter. After 3–4 days of monolayer differentiation, KhES1-derived motor neurons were infected with the *HB9*^*e438*^::*Venus* reporter lentivirus and were observed to extend Venus^+^ neurites 24 h after lentivirus infection (Fig. 6a). At one, two, or four weeks after lentivirus infection, cells were fixed and subjected to immunocytochemistry to detect HB9 and Venus (using an anti-GFP antibody) (Fig. 6b). Most Venus^+^ cells were also positive for HB9 one week after lentiviral infection (HB9/Venus = 84.1 ± 2.4 %, Fig. 6c), suggesting that the expression of the Venus fluorescent protein accurately reflects HB9 expression. Many cells were also positive for another motor neuron marker, ISL-1, after 1 week (ISL-1/Venus = 39.5 ± 13.2 %, Fig. 6c). Moreover, after 4 weeks of monolayer differentiation, most Venus^+^ cells were also positive for ChAT (ChAT/Venus = 83.4 ± 1.7 %, Fig. 6c). These results suggest that the *HB9*^*e438*^::*Venus* lentivirus reporter allows accurate visualization of live motor neurons derived from hPSCs.

To further confirm the relevance of this reporter, we examined the expression of the *HB9*^*e438*^::*Venus* lentivirus through fluorescence-activated cell sorting (FACS) one week after the infection of 201B7-derived motor neurons. We divided the cells into 4 fractions: a negative fraction (gated by the fluorescence intensity of uninfected controls, gray peak) and 3 positive fractions (green peak), where the positive cells were equally subdivided into low-positive, middle-positive, and high-positive fractions (Fig. 6d). The cells in each of the 4 fractions were sorted and subjected to quantitative RT-PCR analysis to detect the expression of *Venus* mRNA and the motor neuron markers *HB9*, *ISL*-*1*, and *ChAT* (Fig. 6e). We observed a significant increase in the expression of *Venus*, *HB9*, and *ISL*-*1* in the high-positive fraction (*p* < *0.05*, Kruskal-Wallis test) and a tendency for the expression of *ChAT* to increase. The expression levels of *HB9* and *ISL*-*1* in the high-positive fraction were 12.4 ± 5.4-fold and 6.4 ± 2.6-fold higher than in the negative fraction. We also examined the expression of Venus fluorescent protein from a reporter lentivirus containing the same vector backbone, but without the *HB9*^*e438*^ enhancer element (background reporter lentivirus; Yellow peak in Fig. 6d), and found that the fluorescence observed in the high-positive fraction of *HB9*^*e438*^::*Venus* reporter-transduced cells overlapped minimally with the fluorescence generated by the background reporter. Together, these results suggested that the high-positive fraction was a *bona fide* “motor neuron fraction” and that this approach can be used for the purification of hPSC-derived motor neurons.

## Discussion

### Rapid and efficient motor neuron induction from hiPSCs

In this report, we have demonstrated a protocol for effective and reproducible motor neuron derivation that does not require transgene expression. Although hESCs and hiPSCs have been differentiated into HB9^+^, ISL-1^+^, and ChAT^+^ motor neurons in several previous studies, our method of motor neuron derivation is superior for the following three reasons: 1) its short culture period and simple culture method; 2) the high purity of the HB9^+^ motor neurons obtained; and 3) its reproducibility among several hPSC lines, thereby minimizing clonal variation. One previously reported method for deriving motor neurons from hPSCs is based on the serum-free floating culture of EB-like aggregates with quick reaggregation (SFEBq) method [2, 16]. Although this method is widely known, it requires 35 days, and the procedure is complicated. Moreover, while the purity of SMI-32^+^ motor neurons derived from 201B7 iPSCs was 35 %, the purity of other iPSC clones was only 15-30 %, with significant variability. Several methods have been established in previous studies to improve the efficiency of motor neuron derivation. For example, a method involving dual SMAD inhibition, using dorsomorphin and SB431542, enabled the production of over 50 % pure HB9^+^ motor neurons, but induction of these motor neurons required approximately 30 days [17, 18]. In other studies, motor neurons were induced within 20–25 days, but the purity of HB9^+^ cells was only 20–30 % [19, 20]. In the present study, we derived 40–50 % pure HB9^+^ motor neurons within 2 weeks, achieving a high efficiency within a short culture period. In addition, the efficiency of motor neuron derivation was consistently 40–50 % among several hPSC clones. Therefore, we have overcome the issue of clonal variation among hiPSC clones [5] and established a more stable *in vitro* model for the pathophysiological analysis of motor neuron diseases.

### GSK3β inhibitor treatment enhances neural induction

In this study, rapid and efficient neural induction was achieved by treating cells with BIO or CHIR99021 in addition to dual SMAD inhibition at an early stage of differentiation. GSK3β inhibitors such as BIO and CHIR99021 have recently been used for neural induction [3, 18, 21]. However, the effects of GSK3β inhibitors have not been discussed in detail. We examined the difference in gene expression following “DS” and “DSB” treatments and found that *PAX6*, *SOX1*, and *NGN2* were upregulated by BIO. β-catenin, which is stabilized by the GSK3β inhibitor BIO, is reported to form a complex with TCF1 and to directly promote the transcription of *PAX6*, *SOX1*, and *NGN2* [22]. In addition, β-catenin is known to form a complex with the intracellular domain of Notch and enhances HES1 expression, which in turn promotes the proliferation of NS/PCs and inhibits the differentiation of NS/PCs [23]. Thus, the activation of Wnt/β-catenin signaling by GSK3β inhibition may directly enhance the transcription of genes associated with the initial steps of neural induction and may also promote the proliferation of induced NS/PCs in our culture system.

### Effect of long-term monolayer culture on the maturation of motor neurons

Cell maturation is particularly important in pathological models of late-onset disease because the cells derived from hPSCs are often immature and do not recapitulate disease phenotypes. In one previous report, more than 100 days of monolayer differentiation were required to detect an apparent phenotype in neurons derived from disease-specific hiPSCs [24]. To recapitulate aging in iPSC-derived neurons, forced expression of Progerin, which is known to enhance cellular aging, was performed to detect the phenotypes of the diseased neurons [25]. Moreover, the recapitulation of cellular stress *in vivo* using glutamate or endoplasmic reticulum (ER) stress driven by tunicamycin has been employed to examine the pathology of neurons or glial cells derived from disease-specific hiPSCs [26, 27]. By contrast, a detailed time-course analysis of gene expression during motor neuron differentiation and the relationship between the length of the culture period and the maturation of motor neurons remains to be reported. We found that the expression of the ChAT protein was upregulated, and the soma of motor neurons became larger and was more clearly stained for ChAT over the course of four weeks of monolayer differentiation, suggesting that motor neurons matured over this time *in vitro* [28]. Thus, a 4 week period of monolayer differentiation is required for the maturation of hPSC-derived motor neurons. Further improvements of culture conditions for maturing motor neurons are expected in future studies.

In this study, we found that HB9 and ChAT protein were unchanged or upregulated, though *HB9* mRNA and *ChAT* mRNA was decreased after 2 weeks of monolayer differentiation. This discrepancy may be attributable to post-transcriptional regulations of these genes during the maturation of motor neurons. In the process of neural differentiation, post-transcriptional regulations are known to play important roles to determine cell fates or behaviors. For instance, ubiquitin specific protease 7 (USP7) deubiquitinates RE1-silencing transcription factor (REST) protein and maintain its protein expression to keep stemness of NS/PCs [29]. For another example, the translation of *FoxP1* in motor neuron progenitors in chick is suppressed by miR-9 and promotes their differentiation into the neurons in medium motor columns via the increase of Hb9 and Lhx3 and the decrease of Isl-1 [30]. In the developing human neocortex, the translation of *Nitric oxide synthase 1* (*NOS1*) mRNA is temporally and regionally regulated by the expression of Fragile X mental retardation protein (FMRP), which is expressed only in pyramidal neurons during early synaptogenesis, though *NOS1* mRNA is widely expressed [31]. Moreover, in the axonal terminal of mature neurons, the expression of Deleted in colorectal carcinoma (DCC) proteins depends on the regulation of local translation to quickly respond to exogenous signals [32]. Considering these region- and stage-specific post-transcriptional regulations, the mismatch between mRNA and protein expression of HB9 and ChAT in the present study may be attributable to such post-transcriptional regulations, which may have important roles in the differentiation or the maturation of PSC-derived motor neurons.

In contrast to the sustained expressions of HB9 and ChAT proteins, both *ISL*-*1* mRNA and ISL-1 protein were significantly decreased after 2 weeks of monolayer differentiation, but the number of ISL-1^+^ cells was not significantly decreased (Fig. 2b). Considering the reproducibility of these results in several hPSC clones, KhES1, 201B7, TIGE-9, and YFE-16 (Figs. 2e, 4e), these results suggest that the expression of ISL-1 per motor neurons decreases as the maturation progressed. Because precise expression of ISL-1 during the maturation of motor neurons has never been discussed in detail so far, the regulation and the biological significance of the expression of ISL-1 should be clarified in the future.

### The *HB9*^*e438*^::*Venus* reporter lentivirus is a useful fluorescent probe for visualizing and purifying HB9^+^ motor neurons

hPSC-derived motor neurons are usually mixed with non-motor neurons following differentiation. They must therefore be identified and purified prior to the examination of motor neuron-specific pathogenesis. For the analysis of multiple hiPSC clones for the purpose of reproducibility, direct labeling of hPSC-derived motor neurons by a lentiviral reporter is an easy and efficient method. In an initial report, 9 kb of the 5′ upstream region of the *HB9* gene, including the *HB9* enhancer/promoter element, was identified and has since been widely used to visualize HB9^+^ motor neurons [33]. Subsequently, it was determined that the 5′ 3.6 kb fragment of this 9 kb element is necessary and sufficient for the transcription of *HB9* in mice, especially the 438-bp or 125-bp enhancer elements containing Pdx and Hox binding domains [15]. Therefore, to construct an efficient reporter system for *HB9*, we generated a reporter lentivirus that expresses the Venus fluorescent protein under the control of the 438-bp *HB9* enhancer element and the *β*-*globin* minimal promoter (*HB9*^*e438*^::*Venus*), which has been shown to specifically label hPSC-derived HB9^+^ motor neurons with more than 80 % accuracy via immunocytochemistry (Fig. 6c). Although this reporter system exhibits some background signal, possibly due to the background activity of the *β*-*globin* minimal promoter, gating our analysis to the high-positive fraction, consisting of the top 1/3 fraction of HB9::Venus^+^ cells, enabled us to obtain *bona fide* HB9^+^ motor neurons (Fig. 6d). Thus, this reporter system allows accurate visualization and purification of hPSC-derived motor neurons and permits us to perform motor neuron-specific analysis, yielding a powerful tool for the pathophysiological analysis of disease-specific motor neurons.

## Conclusions

We established a rapid, efficient and simple method for motor neuron differentiation from hPSCs. We also visualized motor neurons using an HB9 reporter lentivirus (*HB9*^*e438*^::*Venus*). These techniques can be applied to the effective pathophysiological analysis of motor neuron diseases using disease-specific iPSCs.

## Materials and methods

### Isolation of human skin fibroblasts and generation of hiPSCs

Human dermal fibroblasts (HDFs) were obtained from a 24-year-old Japanese male via skin punch biopsy (YF-HDF) and from a 36-year-old Japanese male from the Japanese Collection of Research Bioresources (JCRB) Cell Bank (TIG114-HDF). HDFs were cultured in DMEM (Sigma-Aldrich, St Louis, MO, USA), 10 % fetal bovine serum (FBS; Sigma-Aldrich, St Louis, MO, USA), 2 mM L-glutamine, and 1 % penicillin/streptomycin. Then, 6×10^5^ HDFs were transfected with 1 μg of each of pCXLE-hOCT3/4-shp53 (*OCT4* and *shTP53*), pCXLE-hSK (*SOX2* and *KLF4*) and pCXLE-hUL (*L*-*MYC* and *LIN28*; a gift from Dr. Yamanaka) [34]. Plasmid transfection was performed using the Neon transfection system (Thermo Fisher Scientific, USA). After 6 days, the cells were harvested and plated on mitomycin-C-treated SNL murine fibroblast feeder cells in 0.1 % gelatin-coated tissue culture dishes in human fibroblast medium. On the next day, the medium was changed to standard hESC medium containing DMEM/F-12 (Wako, Japan), 20 % knock out serum replacement (KSR) (Thermo Fisher Scientific, USA), 2 mM L-glutamine, 1 % non-essential amino acids (NEAA) (Sigma-Aldrich, USA), 0.1 mM 2-melchaptethanol (2-ME) (Sigma-Aldrich, USA), 0.5 % penicillin/streptomycin and 4 ng/mL recombinant human fibroblast growth factor-2 (FGF-2) (Peprotech, USA). When the colonies were sufficiently large in size, they were picked and expanded in the same way as hESCs. The properties of the established hiPSC clones were evaluated as described previously (Additional file 1: Figure S1) [34]. hiPSC clones YFE-16 and TIGE-9 were established from YF-HDF and TIG114-HDF, respectively.

All of the experimental procedures for hiPSC production were approved by the ethics committee of the Keio University School of Medicine (approval number 20080016) and the ethics committee of the Aichi Medical University School of Medicine (approval number 14–004). All of the experimental procedures for human embryonic stem cell culture were approved by the ethics committee of the Keio University School of Medicine (approval number 2002–001).

### hESC and hiPSC culture and differentiation *in vitro*

KhES1 (a gift from Dr. Norio Nakatsuji) [9], 201B7 (a gift from Dr. Shinya Yamanaka) [1], TIGE-9, and YFE-16 cells were maintained on mitomycin-C-treated SNL murine fibroblast feeder cells or mouse embryonic fibroblasts (MEFs) in 0.1 % gelatin-coated tissue culture dishes in hESC medium and were used for motor neuron induction. For differentiation, hESC/hiPSC colonies were detached using a dissociation solution containing 0.25 % trypsin, 100 μg/ml collagenase IV (Invitrogen, 17104–019), 1 mM CaCl_2_, and 20 % KSR and cultured in suspension in bacteriological dishes in standard hESC medium without FGF-2, after the removal of SNL feeder cells, with incubation for 1–2 h in gelatin-coated dishes. On day 1, the medium was changed to human embryoid body (hEB) medium containing DMEM/F-12, 5 % KSR, 2 mM L-glutamine, 1 % NEAA, and 0.1 mM 2-ME with 3 μM dorsomorphin dihydrochloride (Santa Cruz, USA), 3 μM SB431542 (Santa Cruz, USA), and 3 μM BIO (Sigma-Aldrich, USA). On day 2, the medium was changed to new hEB medium containing 3 μM dorsomorphin dihydrochloride, 3 μM SB431542, 3 μM BIO, and 1 μM retinoic acid (RA) (Sigma-Aldrich, USA). From day 4 to day 14, hEBs were cultured in hEB medium containing 1 μM RA and 1 μM purmorphamine (Calbiochem, Germany), and the medium was changed every 2–3 days. On day 14, hEBs were enzymatically dissociated into single cells using TrypLE Select (Thermo Fisher Scientific, USA). The dissociated cells were plated on recombinant mouse Laminin (Thermo Fisher Scientific, USA)-coated dishes at a density of 5×10^4^–1×10^5^ cells/cm^2^ and cultured in motor neuron medium (MNM) consisting of media hormone mix (MHM) or KBM Neural Stem medium (Kohjin Bio, Japan) [6] supplemented with 2 % B27 supplement (Thermo Fisher Scientific, USA), 1 % NEAA, 50 nM RA, 500 nM purmorphamine, 10 μM cyclic AMP (cAMP) (Sigma-Aldrich, USA), 10 ng/mL recombinant BDNF (R&D systems, USA), 10 ng/mL recombinant GDNF (R&D systems, USA), 10 ng/mL recombinant human IGF-1 (R&D systems, USA) and 200 ng/mL L-ascorbic acid (Sigma-Aldrich, USA) for 1, 2, or 4 weeks (i.e., 21, 28, or 42 days from EB formation). Half of the medium was changed every 2–3 days.

### Generation of the *HB9*^*e438*^::*Venus* lentivirus and visualization of HB9^+^ motor neurons

HEK293T cells cultured in the Freestyle 293 expression medium (Thermo Fisher Scientific, USA) in 150 mm dishes were transfected with 30 μg of pSIN2-*HB9*^*e438*^-βglo-Venus and 20 μg of each of two packaging vectors (pCMV-VSV-G and pCAG-HIV-gp, kindly provided by Dr. Hiroyuki Miyoshi) in 280 μL of Gene Juice (Novagen, USA), and the medium was changed the next day. Three days after the medium change, the culture supernatant was harvested and centrifuged at 25,000 rpm for 90 min at 4 °C in an Optima LE-80 K ultracentrifuge (Beckman Coulter, USA). After discarding the supernatant, 80 μL of PBS/150 mm dish was added to the pellet, which was resuspended by repeated pipetting to obtain the *HB9*^*e438*^::*Venus* reporter lentivirus.

Lentiviral infection was performed on day 3 or 4 of monolayer motor neuron differentiation. For lentiviral infection, *HB9*^*e438*^::*Venus* in Opti-MEM (Thermo Fisher Scientific, USA) was added to a motor neuron culture, followed by incubation for 2 h, after which the total medium was changed to MNM. The cells were cultured for 1, 2, or 4 weeks (i.e., 24, 31, or 45 days after EB formation) and used for subsequent analyses.

### Hu5/E18 culture, differentiation and co-culture with motor neurons

The human myoblast cell line Hu5/E18 was cultured in collagen type I-coated 10-cm dishes (Iwaki, Japan) or Cellmatrix type I-P (Nitta gelatin, Japan) -coated dishes in myoblast medium containing DMEM, 20 % FBS, 2 mM L-glutamine, 0.5 % penicillin/streptomycin and 2 % Ultroser G serum substitute (Pall, France). For *in vitro* neuro-muscular co-culture, Hu5/E18 cells were plated at a density of 1×10^3^ cells/ml on a Cellmatrix type I-P-coated micro cover glass (Matsunami, Japan) and cultured until the cells reached approximately 50 % confluence. Then, the medium was changed to MNM, and the cells were cultured for 4 additional days, until the formation of myotubes. Partially dissociated EBs derived from KhES1 cells were plated onto Hu5/E18-derived myotubes at a density of 2.5×10^2^–5×10^3^ cells/cm^2^ and co-cultured with the myotubes for 3–4 days in MNM.

### RNA isolation and quantitative RT-PCR analysis

RNA was isolated using an RNeasy mini kit (Qiagen, Germany) and then converted into cDNA using SuperScript III reverse transcriptase (Thermo Fisher Scientific, USA) and Oligo dT primers as described previously [35]. Quantitative RT-PCR analysis was performed using SYBR premix ex Taq II (Takara, Japan) and the Mx3000P (Stratagene, USA), Step One Plus, or ViiA7 real-time PCR system (Applied Biosystems, USA). The amount of cDNA was normalized to that of human-specific *β*-*ACTIN* mRNA. The sequences of the primers and the corresponding cycling conditions are described in Table 1.

**Table 1:** Primer sequences and cycling conditions for quantitative RT-PCR analysis

Gene	Sense		Antisense		Product size	Temp.	10 % DMSO
SOX1	hSOC1-S3755	GAAATAGCCAATGCCAGGTG	hSOX1-AS4039	CCGTGAATACGATGAGTGTTACC	285	62	-
PAX6	hPAX6-S421	TAGGGGCGCGCAGATGTGTG	hPAX6-AS538	TGCATGCTGGCTCTGGCTGG	118	68	-
OLIG2	hOLIG2-S49	GTTCTCCCCTGAGGCTTTTC	hOLIG2-AS223	AGAAAAAGGTCATCGGGCTC	175	67	-
NKX2.2	hNKX2.2-S434	GCTCTGTGGCCGAAGGTCCG	hNKX2.2-AS657	GCTTGAGTCCTGAGGGGGCG	224	62	-
ISL-1	hISL1-S1294	AGCAGCCCAATGACAAAACT	hISL1-AS1494	CTGAAAAATTGACCAGTTGCTG	201	60	-
HB9	hHB9-S951	GTCCACCGCGGGCATGATCC	hHB9-AS1162	TCTTCACCTGGGTCTCGGTGAGC	212	62	-
CHAT	hChAT-S1360	GGAGGCGTGGAGCTCAGCGACACC	hChAT-AS1592	CGGGGAGCTCGCTGACGGAGTCTG	256	62	-
NGN2	hNGN2-S93	AGACACGCACCACCACCACAACAC	hNGN2-AS293	TGACTTTGGCCTGTGCCGGGAATC	201	60	+
EBNA-1 tg	EBNA-1-S	ATCAGGGCCAAGACATAGAGATG	EBNA-1-AS	GCCAATGCAACTTGGACGTT	61	60	-
NANOG total	hNANOG-S286	CCTATGCCTGTGATTTGTGG	hNANOG-AS503	TGTTTCTTGACTGGGACCTTG	218	62	-
OCT4 total	hPOU5F1-S1165	GACAGGGGGAGGGGAGGAGCTAGG	hPOU5F1-AS1283	CTTCCCTCCAACCAGTTGCCCCAAAC	144	62	-
OCT4 tg	hPOU5F1-pla-S	CATTCAAACTGAGGTAAGGG	hPOU5F1-pla-AS	TAGCGTAAAAGGAGCAACATAG	124	62	-
SOX2 tg	hSOX2-pla-S	TTCACATGTCCCAGCACTACCAGA	hSOX2-pla-AS	TTTGTTTGACAGGAGCGACAAT	111	62	-
KLF4 tg	hKLF4-pla-S	CCACCTCGCCTTACACATGAAGA	hKLF4-pla-AS	TAGCGTAAAAGGAGCAACATAG	156	62	-
L-MYC tg	hL-MYC-pla-S	GGCTGAGAAGAGGATGGCTAC	hL-MYC-pla-AS	TTTGTTTGACAGGAGCGACAAT	122	62	-
LIN28 tg	hLIN28-pla-S	AGCCATATGGTAGCCTCATGTCCGC	hLIN28-pla-AS	TAGCGTAAAAGGAGCAACATAG	251	62	-
β-ACTIN (human specific)	hb-ACTIN-S1062	GATCAAGATCATTGCTCCTCCT	hb-ACTIN-AS1241	GGGTGTAACGCAACTAAGTCA	180	62	-

### Immunocytochemistry

Cells were fixed in 4 % paraformaldehyde for 15–25 min at room temperature. After blocking in blocking buffer (PBS containing 5 FBS and 0.3 % Triton X-100), the cells were incubated with primary antibodies overnight at 4 °C. The cells were then rinsed with PBS three times and incubated with Alexa 488-, Alexa 555-, or Alexa 647-conjugated secondary antibodies (Thermo) for 1 h at room temperature. Nuclei were stained with 10 μg/ml Hoechst 33258 (Sigma-Aldrich, USA). The cells were then rinsed with PBS three times, mounted on slides and examined using a universal fluorescence microscope (Axiophoto, Carl Zeiss, Germany) or confocal laser-scanning microscope (LSM700, Carl Zeiss, Germany). The primary antibodies used in these analyses were as follows: HB9 (mouse IgG_1_, 1:200, Developmental Studies Hybridoma Bank [DSHB], USA), ISL-1 (mouse IgG_2a_, 1:200, DSHB, USA), βIII-Tubulin (mouse IgG_2b_, 1:4000, Sigma-Aldrich, USA or rabbit IgG, 1:4000, Biolegend, USA), ChAT (goat IgG, 1:200, Millipore, USA), GFP (rabbit IgG, 1:200, MBL, USA), and MyHC (mouse IgG_2b_, 1:200, DSHB, USA). For the observation of n-AChR clusterization, Alexa fluor 555-conjugated α-Bungarotoxin, (1 μg/mL, Invitrogen, USA) was added to samples and incubated for 1 h.

### Western blot analysis

Western blot analysis was performed as previously reported. A protein sample from a total cell extract (10 μg) was run on 10 % SDS-PAGE and transferred to a nitrocellulose membrane. The blot was then probed with the following primary antibodies: β-ACTIN (mouse IgG_1_, 1:5000, Sigma-Aldrich, USA), HB9 (mouse IgG_1_, 1:2000, DSHB, USA), ISL-1 (mouse IgG_2a_, 1:2000, DSHB, USA), and ChAT (goat IgG, 1:2000, Chemicon, USA). Signals were detected with HRP-conjugated secondary antibodies (Jackson ImmunoResearch laboratory Inc., USA) using an ECL Prime kit (Amersham Biosciences, USA). Quantitative analysis was performed using LAS4000 (Amersham biosciences, USA) and ImageJ software. The amount of protein loaded in each slot was normalized to β-ACTIN.

### Flow cytometry

For flow cytometric analysis, 201B7-derived motor neurons were harvested 7 days after infection with the *HB9*^*e438*^::*Venus* lentivirus (10–11 days of monolayer differentiation) using TrypLE Select. The dissociated cells (5×10^4^–1×10^5^ cells) were suspended in 50–100 μl of Hanks’ balanced salt solution (HBSS) (Thermo Fisher Scientific, USA) containing 2 % fetal bovine serum, 10 mM HEPES, and 1 μg/ml propidium iodide. The cells were then analyzed and sorted based on the expression of the *HB9*^*e438*^::*Venus* reporter using a FACS Aria III cell sorter (BD Biosciences, USA).

### Teratoma formation assay

TIGE-9 and YFE-16 iPSCs were harvested in dissociation solution, collected into tubes, and centrifuged, and the resulting pellets were suspended in hESC medium with 10 μΜ Y-27632 (Wako, Japan), a Rho-associated coiled-coil forming kinase (ROCK) inhibitor. Then, 1×10^5^–5×10^5^ cells were injected into the testes of NOD/SCID mice (Charles River, USA). At 8–10 weeks after injection, the tumors were dissected and fixed with PBS containing 4 % PFA. Paraffin-embedded tissue was sliced and stained with hematoxylin and eosin.

## Additional file

Additional file 1: Figure S1. Evaluation of hiPSCs, TIGE-9 and YFE-16 cells. **a** The genomic integration of episomal vectors was detected through quantitative genomic PCR with primers specific for *EBNA*-*1. EBNA*-*1* copy numbers are normalized to the *β*-*ACTIN* copy number. The *EBNA*-*1* copy numbers were presented as the relative copy numbers to those in fibroblasts transfected with three plasmid vectors for reprogramming and cultured for 6 days (fibroblast-TF day6) (see Materials and methods). **b** Transgene expression in established hiPSC clones was examined via quantitative RT-PCR and is presented as the copy number normalized to that of *β*-*ACTIN*. The expressions of indicated genes are presented as the relative expressions to those in fibroblast-TF day6. **c** The expression levels of the pluripotency markers *OCT4* and *NANOG* in established hiPSC clones were determined through quantitative RT-PCR analysis. **d** Immunocytochemical analysis of TIGE-9 and YFE-16 hiPSCs for OCT4, NANOG, Tra-1–60, and SSEA-4. Scale bar, 300 μm. **e** Hematoxylin and eosin staining of teratomas derived from TIGE-9 and YFE-16 hiPSCs. Scale bar, 200 μm. f Karyotype analysis of TIGE-9 and YFE-16 hiPSCs via G-banding analysis. (PNG 5285 kb)

## Abbreviations

hPSC: Human pluripotent stem cell
hiPSC: Human induced pluripotent stem cell
hESC: Human embryonic stem cell
EB: Embryoid body
