# Supplementary figures and images for: Rapid, efficient, and simple motor neuron differentiation from human pluripotent stem cells
Source: Mol Brain. 2015 Dec 1;8:79. doi: 10.1186/s13041-015-0172-4 (PMC4666063; doi:10.1186/s13041-015-0172-4)

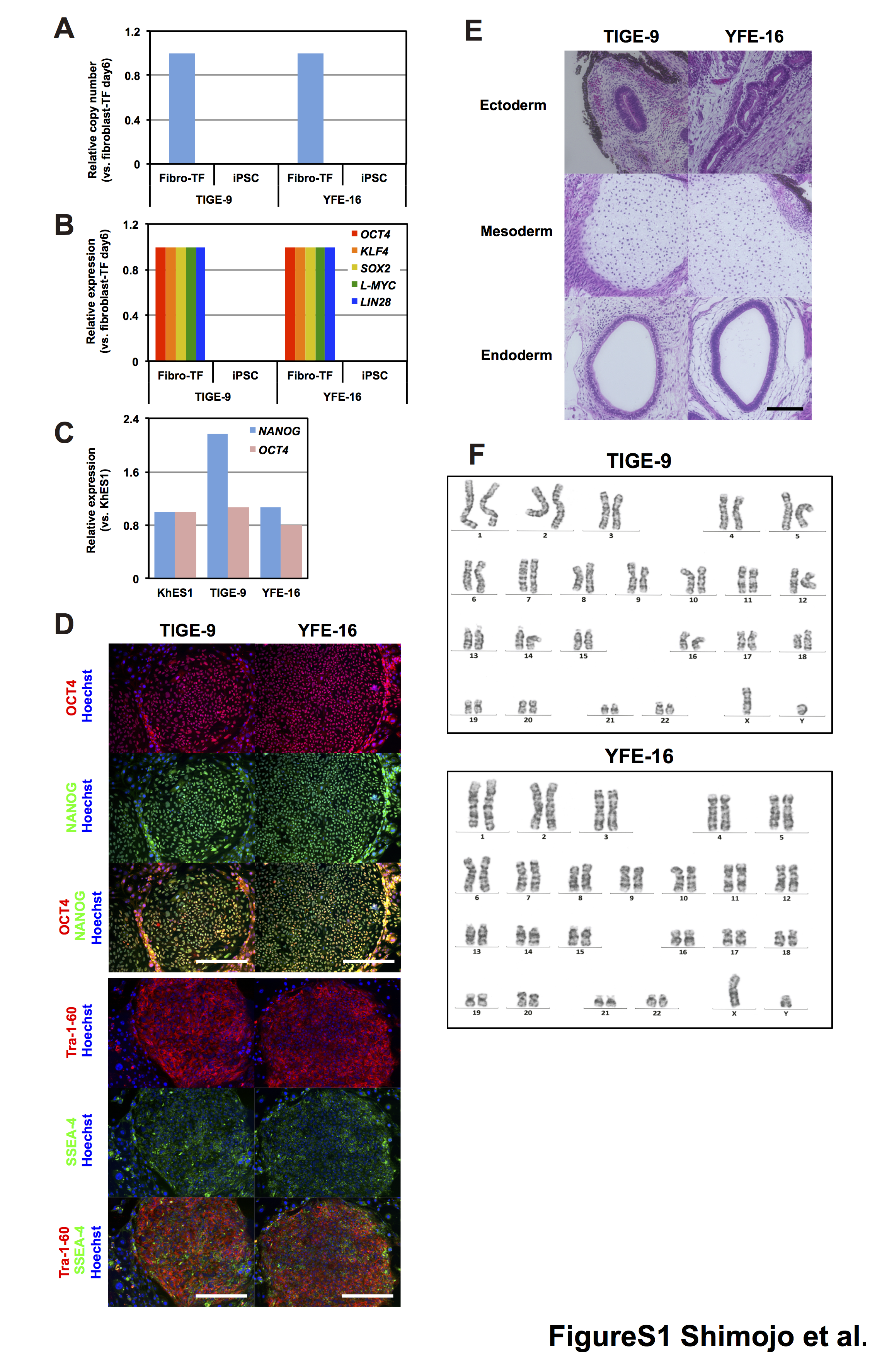

Supplement: Additional file 1: Figure S1. — Evaluation of hiPSCs, TIGE-9 and YFE-16 cells. a The genomic integration of episomal vectors was detected through quantitative genomic PCR with primers specific for EBNA-1. EBNA-1 copy numbers are normalized to the β-ACTIN copy number. The EBNA-1 copy numbers were presented as the relative copy numbers to those in fibroblasts transfected with three plasmid vectors for reprogramming and cultured for 6 days (fibroblast-TF day6) (see Materials and methods). b Transgene expression in established hiPSC clones was examined via quantitative RT-PCR and is presented as the copy number normalized to that of β-ACTIN. The expressions of indicated genes are presented as the relative expressions to those in fibroblast-TF day6. c The expression levels of the pluripotency markers OCT4 and NANOG in established hiPSC clones were determined through quantitative RT-PCR analysis. d Immunocytochemical analysis of TIGE-9 and YFE-16 hiPSCs for OCT4, NANOG, Tra-1–60, and SSEA-4. Scale bar, 300 μm. e Hematoxylin and eosin staining of teratomas derived from TIGE-9 and YFE-16 hiPSCs. Scale bar, 200 μm. f Karyotype analysis of TIGE-9 and YFE-16 hiPSCs via G-banding analysis. (PNG 5285 kb) [file 13041_2015_172_MOESM1_ESM.png]
